# Impact assessment of self-medication on COVID-19 prevalence in Gauteng, South Africa, using an age-structured disease transmission modelling framework

**DOI:** 10.1186/s12889-024-18984-y

**Published:** 2024-06-07

**Authors:** Wisdom S. Avusuglo, Qing Han, Woldegebriel Assefa Woldegerima, Nicola Bragazzi, Ali Asgary, Ali Ahmadi, James Orbinski, Jianhong Wu, Bruce Mellado, Jude Dzevela Kong

**Affiliations:** 1 Africa-Canada Artificial Intelligence and Data Innovation Consortium (ACADIC), York University, Toronto, Canada; 2 The Advanced Disaster, Emergency and Rapid Response Program, York University, Toronto, Canada; 3 Africa-Canada Artificial Intelligence and Data Innovation Consortium (ACADIC), the Dahdaleh Institute for Global Health Research, York University, Toronto, Canada; 4 K. N.Toosi University of Technology, Faculty of Computer Engineering, Tehran, Iran; 5 Africa-Canada Artificial Intelligence and Data Innovation Consortium (ACADIC), University of the Witwatersrand, Johannesburg, South Africa; 6 Artificial Intelligence & Mathematical Modeling Lab (AIMM Lab), Dalla Lana School of Public Health, University of Toronto, Toronto, Canada; 7 Institute of Health Policy, Management and Evaluation (IHPME), University of Toronto, Toronto, Canada; 8 Department of Mathematics, University of Toronto, Toronto, Canada; 9 Global South Artificial Intelligence for Pandemic and Epidemic Preparedness and Response Network (AI4PEP), University of Toronto, Toronto, Canada

**Keywords:** COVID-19, Epidemiology, Self-medication, Age-structured, Disease model

## Abstract

**Objective:**

To assess the impact of self-medication on the transmission dynamics of COVID-19 across different age groups, examine the interplay of vaccination and self-medication in disease spread, and identify the age group most prone to self-medication.

**Methods:**

We developed an age-structured compartmentalized epidemiological model to track the early dynamics of COVID-19. Age-structured data from the Government of Gauteng, encompassing the reported cumulative number of cases and daily confirmed cases, were used to calibrate the model through a Markov Chain Monte Carlo (MCMC) framework. Subsequently, uncertainty and sensitivity analyses were conducted on the model parameters.

**Results:**

We found that self-medication is predominant among the age group 15-64 (74.52%), followed by the age group 0-14 (34.02%), and then the age group 65+ (11.41%). The mean values of the basic reproduction number, the size of the first epidemic peak (the highest magnitude of the disease), and the time of the first epidemic peak (when the first highest magnitude occurs) are 4.16499, 241,715 cases, and 190.376 days, respectively. Moreover, we observed that self-medication among individuals aged 15-64 results in the highest spreading rate of COVID-19 at the onset of the outbreak and has the greatest impact on the first epidemic peak and its timing.

**Conclusion:**

Studies aiming to understand the dynamics of diseases in areas prone to self-medication should account for this practice. There is a need for a campaign against COVID-19-related self-medication, specifically targeting the active population (ages 15-64).

**Supplementary Information:**

The online version contains supplementary material available at 10.1186/s12889-024-18984-y.

## Introduction

In response to the outbreak and alarmingly rapid spread of COVID-19 around the globe, health authorities implemented disease control strategies centered on non-pharmaceutical and pharmaceutical interventions. The effectiveness of these measures is partly dependent on available logistics and individual responses to such interventions. Owing to inadequate health promotion-related resources and limitations in patient health literacy, self-medication and the use of complementary medicine is a common global phenomenon and highly predominant in the global south [1–7]. Evidence of COVID-19-associated self-medication is well documented in the literature (see for example, [8–10] and referenced articles thereof), and the reliance on self-medication by segments of the global population hinders the effectiveness of the various interventions instituted by health authorities. This is because, most intervention measures do not consider the self-medicated population since these cases often go unrecorded, leading to an oversight in the formulation of intervention policies. When measures are implemented without taking into account self-medication, there is a risk of diluting the overall effectiveness of these efforts.

COVID-19-related health policies have benefited from several policy-driven mathematical infectious disease models, where these models have helped shape policy frameworks in the quest to curb the spread of the disease, see, for example, works in [11–16]; also see [17, 18] for a good review on some of these models. Despite the large body of collections of policy-driven mathematical disease models on this subject, there seems to be an inadequate study on mathematical disease model-informed self-medication dynamics. The works in [13, 19] are the few attempts to incorporate the dynamics of self-medication into COVID-19 mathematical models. Both studies show self-medication dynamics has played a major role in the spread of COVID-19, and that efforts should be intensified to put that in check. These works are based on Cameroon and Nigeria COVID-19 cases, respectively. It is imperative that impact of age dynamics is incorporated in the modelling framework in that the literature demonstrates age as an important factor influencing self-medication [9, 20, 21]. Among others, the limitations of the models presented in these studies are that impact of vaccination dynamics on the disease prevalence and age structure of the population were not considered in the modelling framework. In other words, the impact of self-medication across different age groups on the dynamics of the disease transmission, and the interplay of vaccination and self-medication on the spread of the disease were missing.

Self-medication within the context of our proposed study is defined as any approach by an individual to treat the disease through the use of substances (e.g., herbal medicine or over-the-counter drugs) or belief systems (e.g., faith) without consulting a certified professional for such a purpose. These treatments, in most cases, are not efficacious. Not only do they increase the likelihood of prolonged infectious periods of the disease, but they also hinder the isolation of these individuals as they do not make themselves available, thereby increasing the number of infectious individuals in the population. This, in turn, amplifies the force of infection within the population. Therefore, there is a need to incorporate this additional layer of dynamics into the disease modeling framework.

In view of the above, this paper proposes an age-structured mathematical COVID-19 disease model that incorporates self-medication. We considered the case where disease transmission coefficients are different across (age)-groups with associated group specific contacts that map out the mixing pattern within and between these groups. We used case data from Gauteng, South Africa in our study. Gauteng has the largest share of the South African population, having approximately 15.5 million people (26.0%) living in the province [22]. A highly urbanised province having Johannesburg as its capital city. We addressed the following questions: (i) what is the impact of self-medication on the spread and severity of COVID-19 with or without vaccination? This question we address via the impact of the self-medication on the effective reproduction number of COVID-19. (ii) Which of the age groups has the highest incidence of self-medication? We also assessed the sensitivity of the basic reproduction number, first epidemic peak, and first epidemic peak time, respectively, to model parameters (specifically parameters capturing self-medication). We define the first epidemic peak as the first occurrence of the highest magnitude of the disease and the first epidemic peak time refers to the time duration of which we recorded the first highest magnitude of the disease.; the effective reproduction number is the average number of secondary cases per infected individual in the population comprising both susceptible and non-susceptible hosts (in our case, vaccinated individuals) and the basic reproduction number is the effective reproduction number evaluated at the disease-free steady state.

The rest of the paper is organized as follows: The model formulation, related assumptions, and remarks are discussed in “Method” section. The numerical simulations and relevant discussions are provided in “Results” section, where the model is estimated using Gauteng COVID-19 age data, and sensitivity analysis of $\mathcal {R}_0$ (and other model implied quantities) on selected model parameters are also conducted. Finally, the findings are summarized in “Conclusion” section.

## Method

### Model formulation

**Fig. 1:**
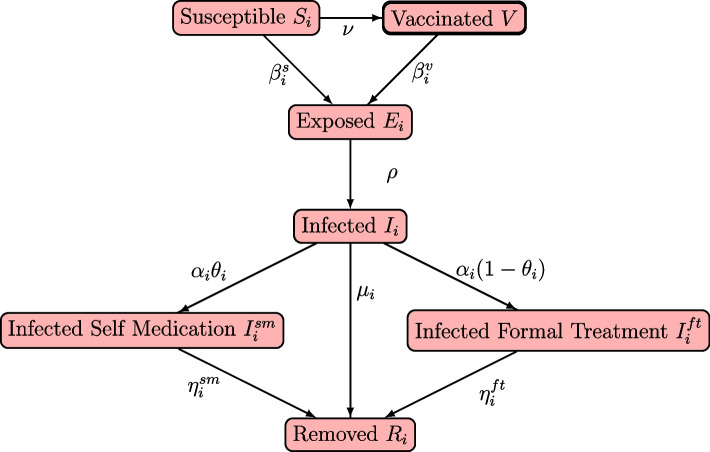
An illustration of COVID-19 transmission dynamics model incorporating self medication and formal treatment

The schematic presentation of the proposed model is given in Fig. 1. The population is stratified into seven compartments: susceptible ($S_i$), vaccinated ($V_i$), exposed ($E_i$), infected ($I_i$), infected self-medication ($I^{sm}_i$), infected formal treatment ($I^{ft}_i$) and removed ($R_i$). Individuals transition across these compartments in accordance with their disease status at each time period. These compartments are further stratified into age groups, for which we denote as *i*.

Susceptible individuals are individuals in the population that are susceptible to the disease; vaccinated are those individuals who have been vaccinated; exposed are those who have exposure to the disease; infected are those who have been infected by the disease and are exhibiting symptoms; infected self-medicated and formal-treatment are those infected individuals who self-medicate and those who seek formal treatment, respectively; removed compartment constitutes recovered individuals—this includes disease induced deaths.

Self-medicated individuals are those who resort to any form of remedy to combat the disease, except using formal treatment. This can take the form of home remedies (examples, traditional or herbal medicines, over-the-counter drugs, etc.), spiritual cleansing or prayers as recorded in some jurisdictions [13, 19], and others. These treatments in most cases are not efficacious; see for instance [23] and references therein. Not only does this increase the likelihood of prolonged infectious periods of the disease, it prevents isolation of these individuals as they do not make themselves available, therefore increasing the number of infectious individuals in the population, which will then amplify the force of infection within the population. consequently, the need to incorporate this additional layer of dynamic into the disease modelling framework. The formal-treatment compartment constitutes individuals who resort to treatment at a certified or government recognized health care space. We define treatment as the administration of drugs, or any other medication by healthcare professionals.

The disease system dynamics are as follows: we assume a short duration of the disease as in the case of a seasonal disease. The assumption of short duration of the disease pertains to the exclusion of demographic parameters and not related to infection parameters; we excluded population demographics such as birth and death rates in the modelling framework. Birth and natural death rates can be excluded from mathematical models when investigating disease dynamics occurring within few weeks or months. See , for example, works in [24–30]. Specifically, the works outlined in [25, 26, 28–30] provided COVID-19 mathematical models excluding effects of birth and natural death rates. Mathematical models without demographic parameters have extensively been used to assess dynamics of disease epidemics. Models of this nature (epidemic models) are used to model rapid outbreaks that happens in less than a year [27].

Now, Observe that subscript *i* on model parameters corresponds to the parameters for each of the age groups and that those without subscript *i* imply that the parameter is the same across the age groups. Against this backdrop, the model assumes a per capita vaccination rate $\nu$ ($\nu S_i$ are vaccinated and enters $V_i$) and that vaccine-induced immunity lasts for the entire disease outbreak period. Here we assume that $\nu$ is the same across all the age groups as we recognize that South Africa’s vaccination program commenced in February 2021 [31], implying the vaccination commencement date for Gauteng Province is not earlier done February 2021. The current study considers COVID-19 infection period between March 1, 2020 and July 5, 2020-there was no vaccination in place nor vaccination strategy. Our considered period is in line with the research questions we want to address: it serves as the base period to carry out sensitivity analysis on the study’s parameters of interest. Also, the proposed study is a generic study, not an empirical study, therefore assuming equal vaccination rates across the different age groups addresses the purpose of our study. We define vaccination under this setting as that which confers protection of individuals from the disease.

Individuals in $S_i$ and $V_i$ are infected with the disease at the respective rates of $\mathcal {B}^s_i$ and $\mathcal {B}^v_i$—we assume that transmission is frequency dependent. $\mathcal {B}^s_i S_i+\mathcal {B}^v_i V_i$ of individuals enter the exposed compartment $E_i$. The latency rate for which individuals transition from $E_i$ is $\rho$. Thus, $\rho E_i$ individuals transition from $E_i$ to the infected compartment $I_i$. We acknowledge that this assumption implies impact of disease transmissions is via $\mathcal {B}^s_i$ and $\mathcal {B}^v_i$.

Following the work in [13], we assume individuals are detected of the disease at the rate $\alpha _i$. Consequently, we assume $\alpha _i \theta _i I_i$ and $\alpha _i(1- \theta _i) I_i$ number of individuals migrates from $I_i$ to $I^{sm}_i$ and $I^{ft}_i$, respectively, where $\theta _i$ is the proportion of those entering $I^{sm}_i$ and $(1-\theta _i)$ entering $I^{ft}_i$. Finally, individuals are respectively removed from $I_i,I^{sm}_i$ and $I^{ft}$ at the rates $\mu _i,\eta ^{sm}_i$ and $\eta ^{ft}_i$. System 1 describes the evolution of the disease across the different compartments and age groups.

1 $$\begin{aligned} \dot{S}_i{} & {} =-\nu S_i- \mathcal {B}^s_i S_i, \nonumber \\ \dot{V}_i{} & {} =\nu S_i- \mathcal {B}^v_i V_i, \nonumber \\ \dot{E}_i{} & {} = \mathcal {B}^s_i S_i+ \mathcal {B}^v_i V_i-\rho E_i, \nonumber \\ \dot{I}_i{} & {} = \rho E_i -\alpha _i I_i-\mu _i I_i, \nonumber \\ \dot{I}^{sm}_i{} & {} =\alpha _i \theta _i I_i - \eta ^{sm}_i I^{sm}_i, \nonumber \\ \dot{I}^{ft}_i{} & {} =\alpha _i (1-\theta _i) I_i -\eta ^{ft}_i I^{ft}_i, \nonumber \\ \dot{R}_i{} & {} =\mu _i I_i +\eta ^{sm}_i I^{sm}_i +\eta ^{ft}_i I^{ft}_i, \end{aligned}$$

with initial condition

$$\begin{aligned} Y(0)=(S_{i}(0),V_{i}(0),E_{i}(0),I^{s}_i(0),I^{sm}_{i}(0), I^{ft}_{i}(0), R_{i}(0))\in \mathbb {R}^+. \end{aligned}$$

#### Force of infection $\mathcal {B}^s_i$ and $\mathcal {B}^v_i$ and reproduction number

Since the underlying framework of the proposed model and study is age structured, the disease force of infection ($\mathcal {B}^s_i$ and $\mathcal {B}^v_i$), defined as the rate at which susceptible/vaccinated individuals become exposed, is group specific; this is influenced by the activities within and between groups, and is captured by the overall contact levels. The intensity of a group’s contact level influences the disease cases within the group and at the population level. Following the work in [32, 33], and related works in the field, we model the force of infection for a representative group as follows: Let $x_{ij}$ be the average number of contacts per person per unit time in a representative group, where $i=j$ is within group contact and $i\ne j$ outside group contacts. The unit time could be day(s) or month(s) (this study considered daily number of contacts). This defines the contact matrix in the population. We assumed heterogeneous effective transmission coefficients across age structures; these are respectively denoted as $\beta ^s_i$ and $\beta ^v_i$ for susceptible and vaccinated individuals. $\mathcal {B}^s_i$ and $\mathcal {B}^v_i$ are expressed as

2 $$\begin{aligned} \mathcal {B}^s_i{} & {} =\sum \limits _{j=1}^n \frac{\beta ^s_i x_{ij} \left( I_j + I^{sm}_j\right) }{N_j}, \nonumber \\ \mathcal {B}^v_i{} & {} =(1-e)\mathcal {B}^s_i, \end{aligned}$$

where we note that

3 $$\begin{aligned} \beta ^{v}_i =(1-e)\beta ^{s}_i. \end{aligned}$$

$N_j$ is the population size of the individuals across age group *j* for all disease compartments, *n* is the number of age groups, and $0\le e \le 1$ captures the vaccine efficacy. Assuming proportionate mixing of individuals between groups. Observe that individuals in the formal treatment compartment are excluded from the expression for the force of infection; this is attributable to the assumption that Individuals in the formal treatment compartment are assumed to receive effective treatment such that their infectivity is reduced to a negligible level. The resulting general effective reproduction number from the model is derived as (See Supplementary Materials)

4 $$\begin{aligned} \mathcal {R}_t=\sum \limits _{i=1}^n x_{i}\left[ \frac{S_{i}(t)\beta _i^s\left( \alpha _i\theta _i+\eta ^{sm}_i\right) +V_{i}(t)\beta _i^v\left( \alpha _i\theta _i+\eta ^{sm}_i\right) }{N_i(\alpha _i+\mu _i)\eta ^{sm}_i}\right] , \end{aligned}$$

where $x_i$ in Eq. 4 is the daily number of contacts made by an individual in group *i* per unit time. We note that the effective reproduction number is the average number of secondary cases per infected individual in the population comprising both susceptible and non-susceptible hosts (in our case, vaccinated individuals). The basic reproduction number is the effective reproduction number evaluated at the disease free steady state. It is the average number of secondary infections produced by an infected individual in the population where everyone is susceptible. Observing Eq. (4) leads to the following remarks.

##### Remark 1

All other parameters held constant, the proportion of individuals who undergo self medication $\theta _i$ positively relates to $\mathcal {R}_t$ and $\mathcal {R}_0$. Implying increasing $\theta _i$ increases $\mathcal {R}_t$ and $\mathcal {R}_0$.

The epidemiological implication of Remark 1 is that the more number of people self-medicate the more average number of secondary cases of the disease at time *t* in the population, thus to reduce the disease spread, a campaign against self-medication may be effective.

##### Remark 2

All other parameters held constant, the detection rate ($\alpha _i$) negatively relates to $\mathcal {R}_t$ and $\mathcal {R}_0$.

##### Proof

The prove of Remark 2 can be shown by observing that the first partial derivative of $\mathcal {R}_t$ (Eq. (4)) with respective to $\alpha _i$ is negative for every value of $\alpha _i$, thus $R_t$ decreases as a function of $\alpha _i$. $\square$

Remark 2 indicates, as a policy implication, increasing the detection rate of the disease can help curb its spread, when other parameters are held constant. Increasing detection rate can reduce disease incidence in the population.

#### Markov Chain Monte Carlo estimation scheme

Markov Chain Monte Carlo Delay Rejection Adaptive Metropolis [34] was used to estimate model parameters. We adopted the Matlab package mcmcrun provided in [35]. The model’s goodness of fit was assessed using the normalized mean square error (NMSE), as found in [13]. The likelihood function of the observed state, the number of new infections, is assumed as normal distribution and the prior distributions of the parameters are assumed as normally distributed. We started the estimation process from non-optimized values; we did three runs of the algorithm, starting from the values of the previous run in order to locate the appropriate posterior distribution of the parameters. Each of the runs has 10,000 simulations, making 30000 simulations in total. We then estimated the mean from the individual final chains of the model parameters of interest.

#### Estimating contact matrix

We employed the approach used in [36–38] in estimating the contact matrix. We partitioned the Gauteng case data into the three age groups: 0-14, 15-64, and 65+. This we did by noting that the case data is partitioned into age groups (0-10, 11-10, 21-30, 31-40, 41-50, 51-60, 61-70, 71-80, 80+); and the age groups do not match appreciably with our proposed age groups for the study. Therefore, we estimated the cases in each age groups (0-14, 15-64, and 65+) by first estimating the number of cases in, for example, the age group 11-14, and then add that estimated number cases to the cases in age group 0-10 to arrive at the number of cases in 0-14. We do same for 15-64, and 65+. As notational example, let $C_{0-10}$ and $C_{11-20}$ be number of cases in age groups 0-10 and 11-20 respectively, $P_{11-14}$ and $P_{11-20}$ be the respective population size of age group 11-14 and 11-20, then the number of cases for the age group 0-14 is given as

$$\begin{aligned} C_{0-14}=C_{11-20} \frac{P_{11-14}}{P_{11-20}}+C_{0-10}. \end{aligned}$$

The estimated number of cases for each age group for our proposed age groups is then used as input in our estimation scheme. Note that, this approach assumes that cases are evenly distributed among the groups.

## Results

### Numerical analysis

This section discusses numerical analyses by first presenting the estimated values of the parameters not found in the literature. We based our estimation procedure on COVID-19 cases in Gauteng, South Africa. Gauteng has the largest share of the South African population, having approximately 15.5 million people (26.0%) living in the province [22]. Table 1 presents the demographic of Gauteng by age range. Observe that age 15-65 constitutes the largest population.

**Table 1:** Gauteng: Population demographics [22]

Age	Total	Age	Total
0-4	1 304 927	50-54	716 093
5-9	1 224 646	55-59	598 836
10-14	1 117 926	60-64	479 181
15-19	1 062 602	65-69	360 126
20-24	1 340 369	70-74	244 621
25-29	1 655 304	70-79	141 871
30-34	1 719 113	80+	84 412
35-39	1 425 916	45-49	908 134
40-44	1 104 058	Total	15 488 137

#### Data set on Gauteng COVID-19 cases

The data set on Gauteng province COVID-19 cases is now publicly available at: https://www.covid19sa.org/. The data set is a record of COVID-19 cases on different disease age groups: 0-10, 11-10, 21-30, 31-40, 41-50, 51-60, 61-70, 71-80, and above 80 (80+). We considered cases for the period spanning between March 1, 2020 and July 5, 2020, inclusive. For the purpose of our study we stratified the population into three age groups: 0-14, 15-64, and above 65 (65+). This stratification is to group the population into active and non-active sub-populations as well as dependent and independent sub-populations. We hereby assume individuals in ages 0-14 are dependent sub-population and those in 15-64 and 65+ are independent with regards to issues relating to self medication; the age group 15-64 is the most active sub-population.

#### Estimated contact matrix for Gauteng

**Fig. 2:**
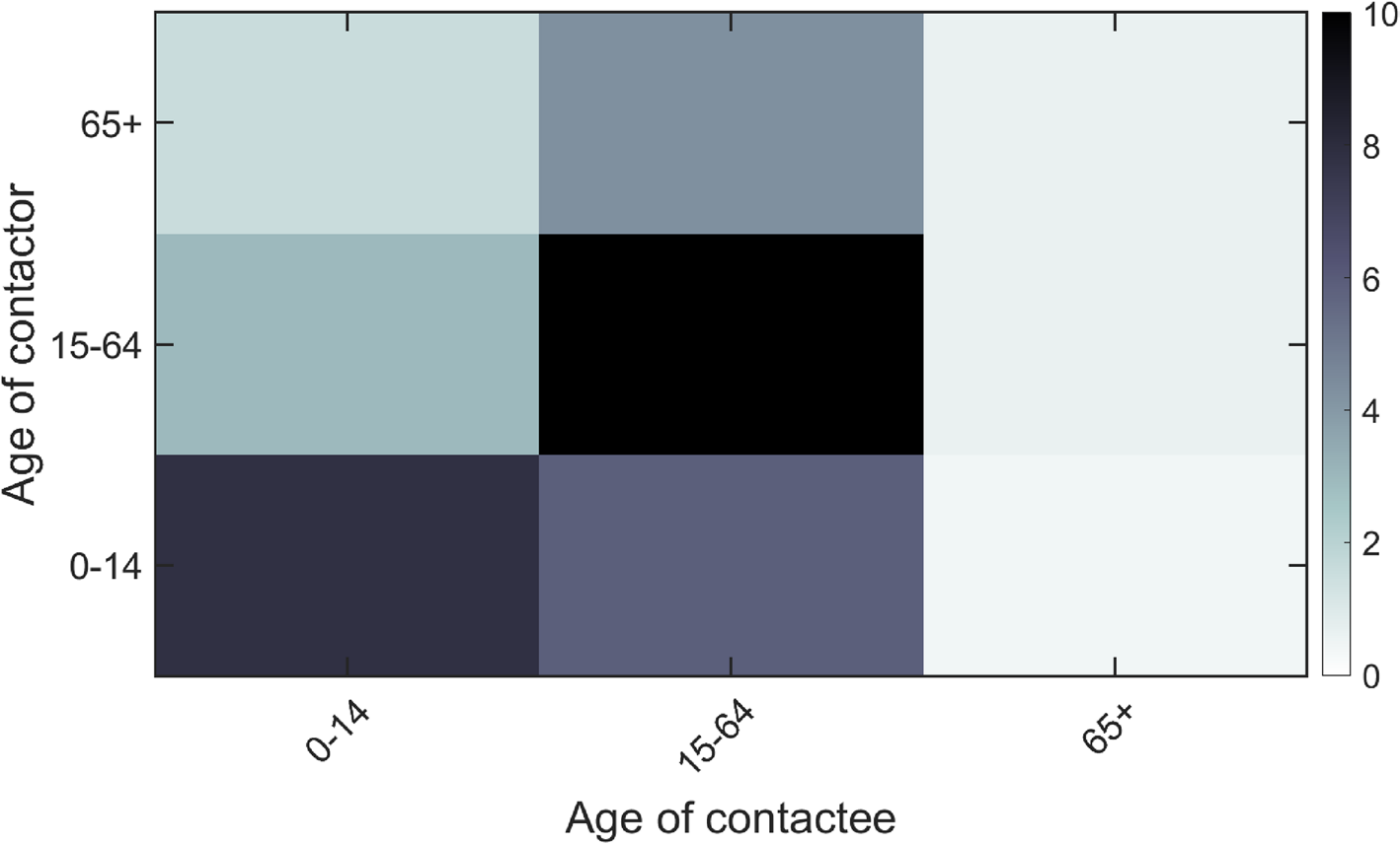
Gauteng Province: Contact matrix illustrating the contacts among population age groups: 0-14, 15-64, and 65+. The color bar indicates the gradation of the average number of contacts by individuals within and between groups per unit time. We assume a static mixing behaviour (that is, number of contacts is static across the disease period. We note that the population sizes of each age group are respectively given as 4710101, 9467823, and 1310211. This corresponds to the Gauteng province mid year 2020 population demography reported in [22]. We used the estimated (synthetic) South African’s population contact matrix provided in [38]

We used South African’s population contact matrix to estimate that of Gauteng province, and is adopted from [38]; we used the synthetic contact matrix estimated in the paper, a decision informed by the fact the estimated contact matrix reflects COVID-19 impact on the population social contact. The contact matrix is estimated as

5 $$\begin{aligned} \text {Contact Matrix}= \left( \begin{array}{lll} 7.8051 &{} 5.8937 &{} 0.4289\\ 2.9320 &{} 12.0201 &{} 0.5901\\ 1.5420 &{} 4.2639 &{} 0.6189 \end{array}\right) , \end{aligned}$$

where we used density correction approach for reciprocity correction. The graphical presentation of the contact matrix is given in Fig. 2. Observe that the Age group 15-64 has the highest average number of within group contacts and age group 65 and above the least.

#### Model parameters estimation and numerical analysis

Recall the age structure Gauteng’s COVID-19 case data is incompatible with the defined age structures for our studies—case data is partitioned into age groups (0-10, 11-10, 21-30, 31-40, 41-50, 51-60, 61-70, 71-80, 80+). Our interest is to group the cases by age groups 0-14, 15-64, and 65 and above. The estimated population age structure of Gauteng is grouped as 0-4, 5-9, 10-14,15-19, 20-24,25-29, 30-34, 35-39, 40-44, 45-49, 50-54, 55-59, 60-64, 65-69, 70-74, 75-79; see [22] for population demographics. This implies we need to estimate the population sizes of the age groups of interest. We first have to transform the COVID-19 case age-structured data from (0-10, 11-10, 21-30, 31-40, 41-50, 51-60, 61-70, 71-80, 80+) to (0-4, 5-9, 10-14,15-19, 20-24,25-29, 30-34, 35-39, 40-44, 45-49, 50-54, 55-59, 60-64, 65-69, 70-74, 75-79). And then group into 0-14, 15-64, and 65 and above. We do this by borrowing the ideas from [39], outlined below:

i. Suppose an observed data points $(x_j,y_j), j=1,2,...N$, where we define $x_j$ in our setting as ages in 5 years intervals and $y_j$, the cumulative population sizes for ages up to and including $x_i$
ii. We can define a function *f*(*x*) that interpolates all points between each of the consecutive pair of knots $x_j$ and $x_{j+1}$.
iii. After estimating the pairs $(x_j, y_j)$, we can then recover individual population estimates for each of the age groups by setting the population estimate for a representative age group $x_j$ as $y_{j+1}-y_{j}$.

**Fig. 3:**
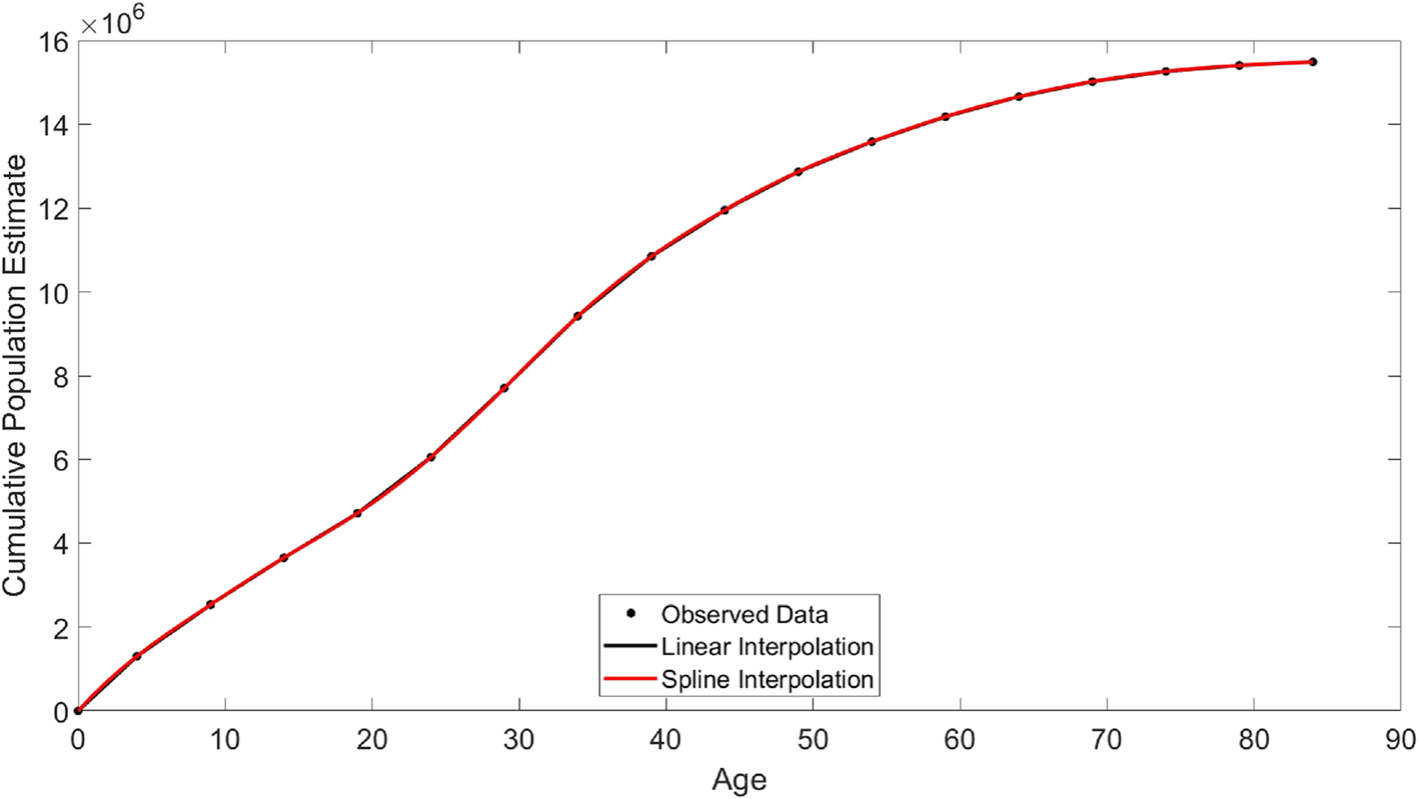
Gauteng Province: Interpolation of cumulative population sizes across ages using Linear and Spline Interpolations. Data source [22]

Figure 3 is the plot of the cumulative curve. It plots the the age and the cumulative population size. The dots in the figure are the cumulative population size obtained from [22]. The solid black and red lines connects the interpolated points (black dots) using the linear and spline interpolation schemes. Observe these interpolation schemes approximately coincide. For this reason, we used the estimated cumulative population sizes derived from the linear interpolation scheme for our analysis. We obtain the population size estimates for the required age groups using the method outlined above.

**Fig. 4:**
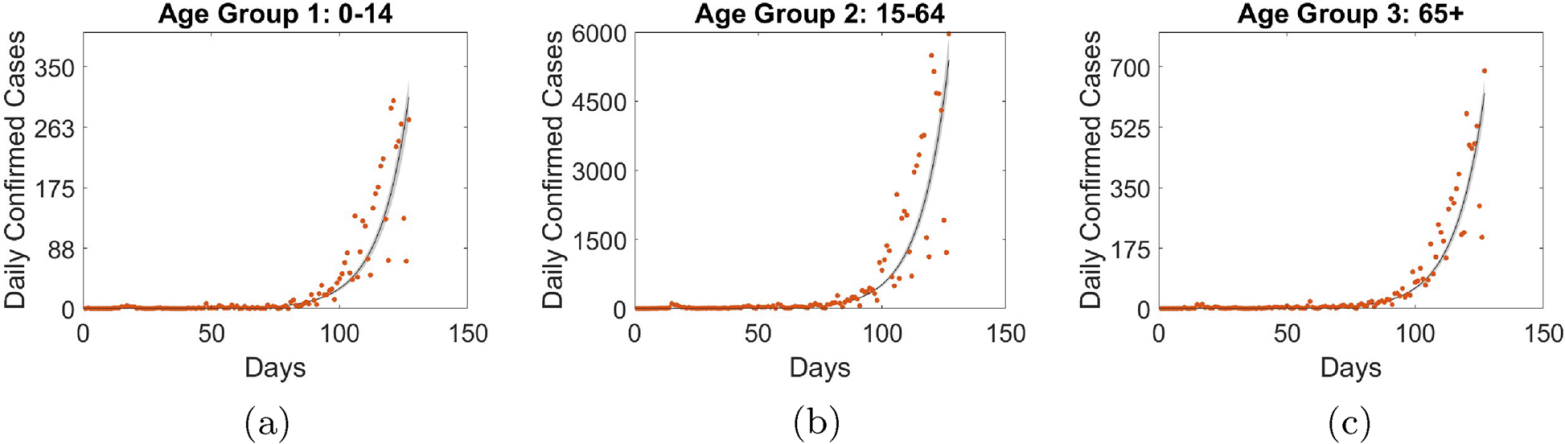
Gauteng Province: Dots represent the observed daily cases and the solid line is the fitted model. The population sizes across the three age groups are respectively given as 4710101, 9467823, and 1310211, for age groups 0-14, 15-64, and 65+. Initial values are presented in Table 2

**Fig. 5:**
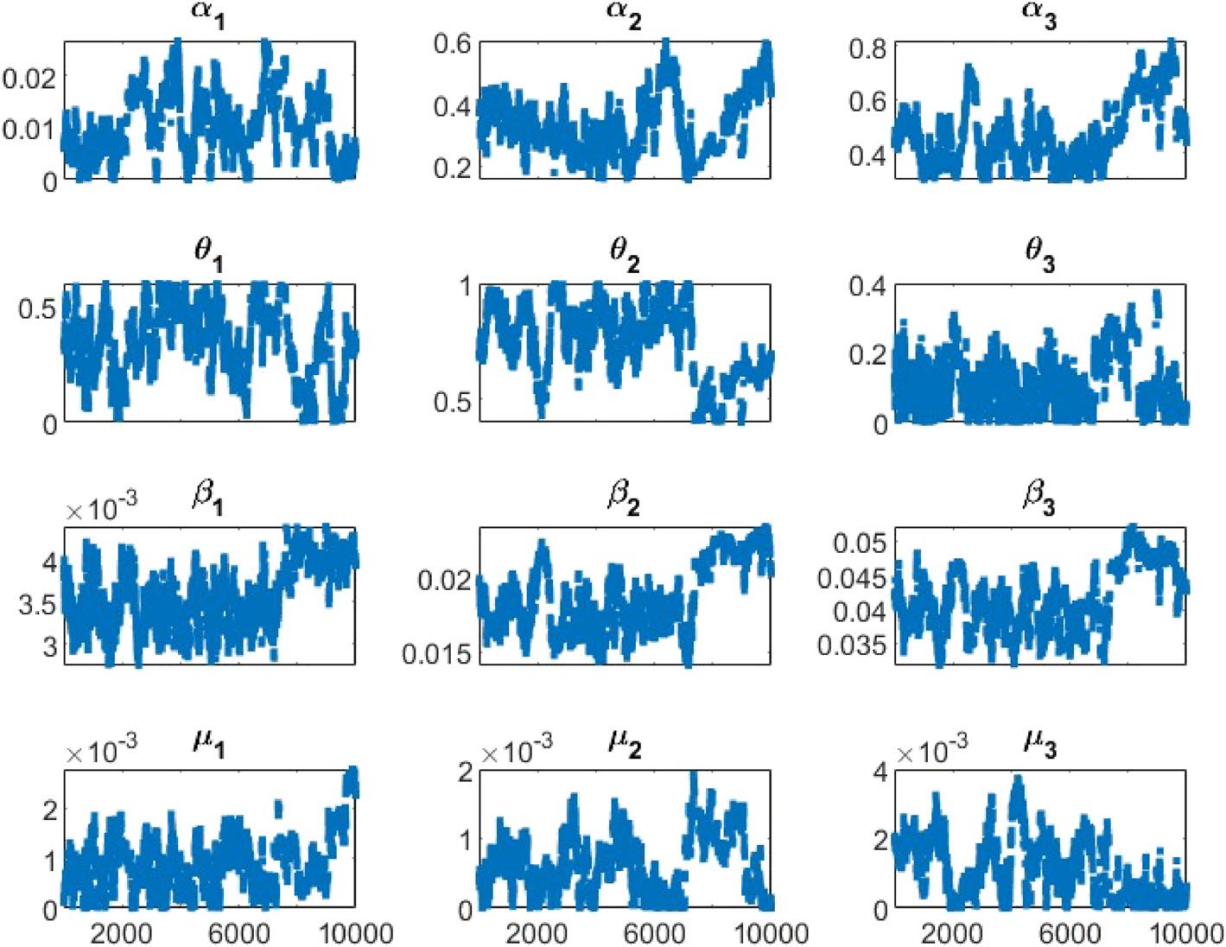
The chain plots of the parameters of interest. The axis is the number of simulations (the third run) and the vertical axis is the generated values of the parameters

Figure 4 presents the plot of the estimated model for the three Age groups, and we see an appreciable fit ($NMSE\approx 72.95\%$). The grey region indicates 95% confidence bands of the estimated disease states, which we obtained by sampling the final respective chains of the parameters and using the resulting sample to calculate the predictive limit. The chain plots are presented in Fig. 5, and it shows generally appreciable convergence of the chains. Table 2 presents the values of model parameters not estimated and initial system state values. We set the value of the measure of vaccine efficacy *e* at 93% (this coincides with that of BNT162b2 (89.0% to 93.2%) [40]). The model implied estimates indicates that self-medication is predominant among Age group 15-64 (74.52%), followed by Age group 0-14 (34.02%); Age group 65+ records 11.41%.

**Table 2:** Gauteng Province: Baseline values for the model parameters

Parameter	Definition	Values	Sources
$\nu _1,\nu _2,\nu _3$	per capita vaccination rate	0	Assumed
$\rho$	Latency rate	1/5.2 day^-1^	[41]
$\eta ^{sm}_1,\eta ^{sm}_2$	Removal rate of self-medicated individuals for age group 0-14 and 15-64	1/14 day^-1^	[13]
$\eta ^{sm}_3$	Removal rate of self-medicated individuals for age group 65+	1/28 day^-1^	[13]
$\eta ^{ft}_1,\eta ^{ft}_2$	Removal rate of individuals individuals who go for formal treatment for age group 0-14 and 15-64	1/14 day^-1^	[13]
$\eta ^{ft}_3$	Removal rate of individuals who go for formal treatment for age group 65+	1/28 day^-1^	[13]
*e*	measure of vaccine efficacy	93%	Assumed
$S_{1}(0)$		4710101	Assumed
$S_{2}(0)$		9467822	Assumed
$S_{3}(0)$		1310211	Assumed
$V_{1}(0), V_{2}(0), V_{3}(0)$		0	Assumed
$E_{1}(0),E_{2}(0),E_{3}(0)$		0	Assumed
$I_{1}(0), I_{3}(0)$		0	Observed data
$I_{2}(0)$		1	Observed data
$I^{sm}_{1}(0),I^{sm}_{2}(0),I^{sm}_{3}(0)$		0	Assumed
$I^{ft}_{1}(0),I^{ft}_{2}(0),I^{ft}_{3}(0)$		0	Assumed
$R_{1}(0), R_{2}(0), R_{3}(0)$		0	Assumed

**Table 3:** Gauteng Province: Estimated model parameters

Parameter	Definitions	Values
$\alpha _1$	Detection rate in age group 0-14	0.010287 day^-1^
$\alpha _2$	Detection rate in age group 15-64	0.33105 day^-1^
$\alpha _3$	Detection rate in age group 65+	0.4751 day^-1^
$\theta _1$	Proportion of self medicated population in age group 0-14	0.34017
$\theta _2$	Proportion of self medicated population in age group 15-64	0.74522
$\theta _3$	Proportion of self medicated population in age group 65+	0.11408
$\beta ^s_1$	Transmission coefficient relating to susceptibles age group 1	0.0035613
$\beta ^s_2$	Transmission coefficient relating to susceptibles age group 2	0.018702
$\beta ^s_3$	Transmission coefficient relating to susceptibles age group 3	0.041503
$\mu _1$	Removal rate for infected age group 0-14	0.00089483 day^-1^
$\mu _2$	Removal rate for infected age group 15-64	0.00061965 day^-1^
$\mu _3$	Removal rate for infected age group 65+	0.0011337 day^-1^

#### Sensitivity analysis

This section discusses sensitivity analysis of the basic reproduction number $\mathcal {R}_0$, first peak magnitude, and first epidemic peak time to model parameters respectively. The derivation of the $\mathcal {R}_0$ is presented in the Supplementary Materials. We employed the Latin Hypercube Sampling Partial Rank Correlation Coefficient (PRCC) scheme [42, 43]. The PRCC is a measure of the strength of a linear association between the model parameters and model derived quantities or outputs (in our case, the $\mathcal {R}_0$, first epidemic peak magnitude, and first epidemic peak time); the value is between $-1$ and $+1$. We assumed a parameter range of values of $\pm 50$ of the values of the parameters of interest, presented in Table 3.

**Fig. 6:**
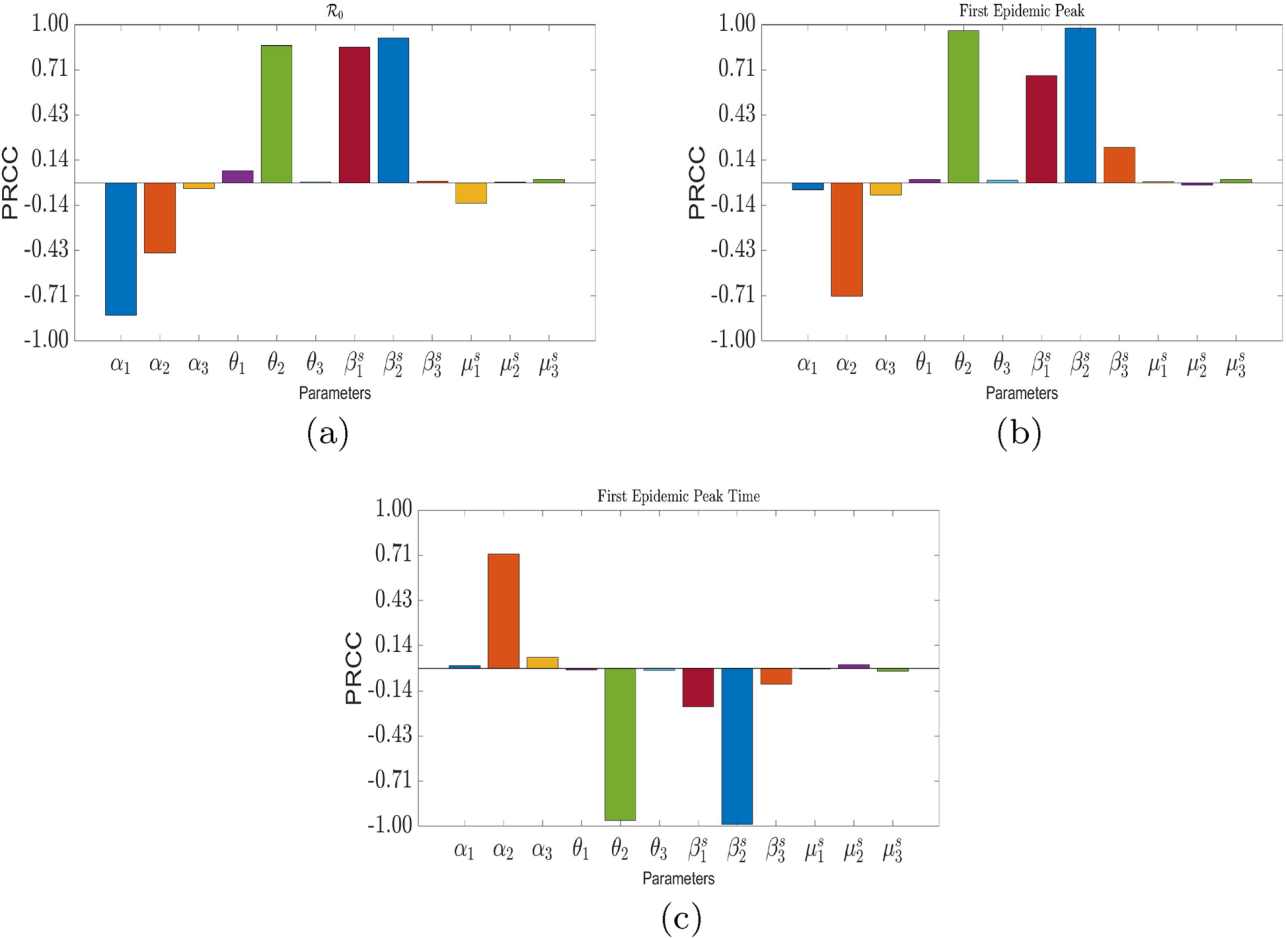
Sensitivity analysis of model quantities to the respective parameters of interest. Baseline parameters and initial state values are found in Tables 2 and 3

Figure 6 is the visualization of the degree of the sensitivity of $\mathcal {R}_0$, first peak epidemic, and first epidemic peak time to selected model parameters . We observed that $\mathcal {R}_0$ has high degree of correlation with $\alpha _1,\theta _2$, $\beta ^s_1$, and $\beta _2^s$–see Fig. 6a; the First epidemic peak of the disease is strongly correlated with $\alpha _2$, $\theta _2$, and $\beta _2^s$—see Fig. 6b; and the first epidemic peak time is strongly correlated with $\alpha _2,\theta _2,\beta ^s_2$. Table 4 present a summary of the above-mentioned observations. In the interest of our study, the policy parameters of interest are the proportions of individuals who self-medicate across the various age groups—$\theta _1,\theta _2$ and $\theta _3$, and vaccination rate $\nu$. We note that $\theta _2$ has the most impact on $\mathcal {R}_0$, First Epidemic Peak, and First Epidemic Peak Time.

**Table 4:** Summary: PRCC sensitivity analysis

	High	Low
$\mathcal {R}_0$	$\alpha _1,\theta _2,\beta ^s_1,\beta _2^s$	$\alpha _2,\alpha _3,\theta _1,\theta _3,\beta _3^s,\mu _1,\mu _2,\mu _3$
First Infection Peak	$\alpha _2,\theta _2$, $\beta _1^s,\beta _2^s$	$\alpha _1, \alpha _3, \theta _1, \theta _3, \beta _3^s, \mu _1,\mu _2,\mu _3$
First Epidemic Peak Time	$\alpha _2,\theta _2,\beta ^s_2$	$\alpha _1,\alpha _3,\theta _1,\theta _3,\beta _1^s,\beta ^s_3,\mu _1,\mu _2,\mu _3$

**Fig. 7:**
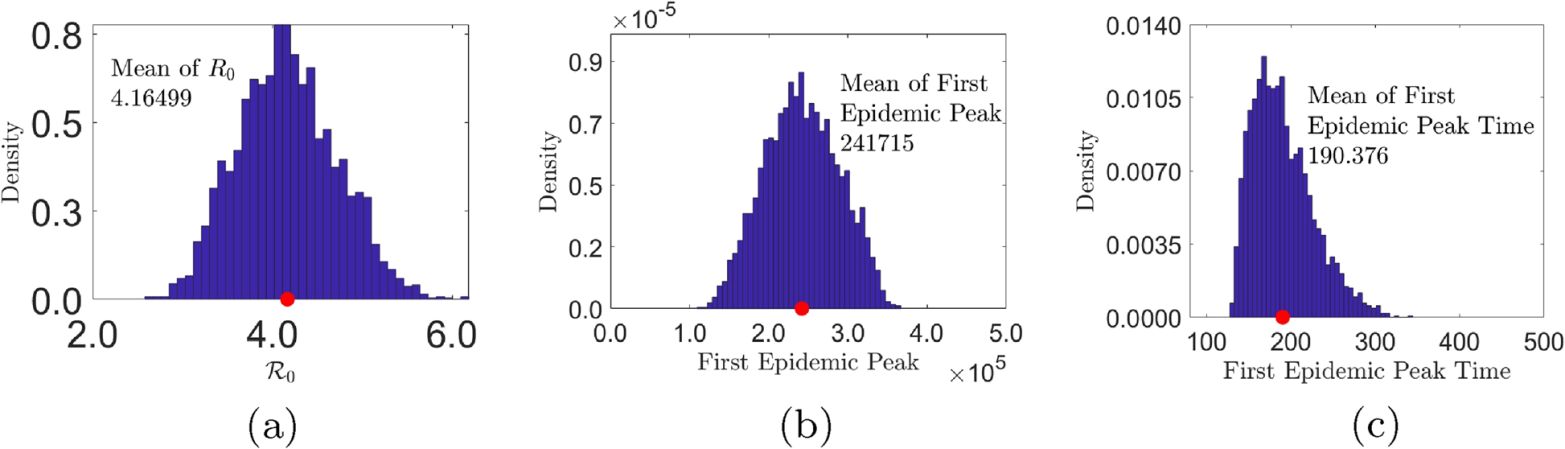
Gauteng Province: Uncertainty Analysis. The baseline parameter values are given in Table 2

Figure 7 presents the respective histograms of $R_0$, First Epidemic Peak, and First Epidemic Peak Time, with their respective means. The average $R_0$ is 4.16499, and that of First Epidemic Peak and First Epidemic Peak Time are 241,715 and 190.375, respectively.

**Fig. 8:**
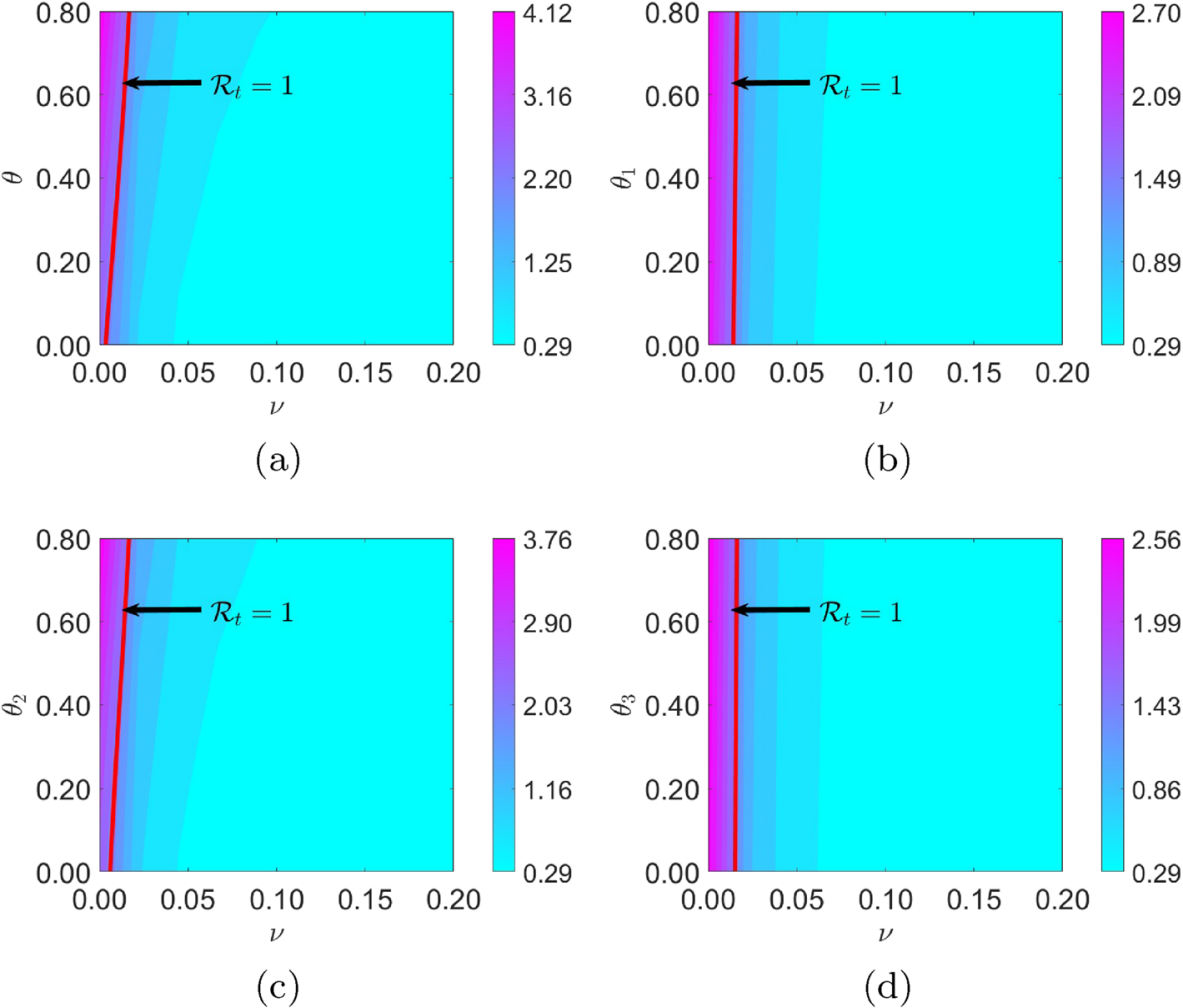
Assessing the impact of the interaction of the proportion of self-medicated individuals $\theta$ and vaccination rate $\nu$ on effective reproduction number $\mathcal {R}_t$. $\mathcal {R_t}$ reported here is the average of the $\mathcal {R}_t$s over the period 0 to 127. Parameter values are given in Tables 2 and 3. The population sizes across the three age groups are respectively given as 4710101, 9467823, and 1310211, for age groups 0-14, 15-64, and 65+. Initial values and base line parameter values are given in Table 2

The contour plots in Fig. 8 demonstrates the joint impact of self-medication and vaccination on the effective reproduction number, and thus the spread of COVID-19. The figure shows that the joint impact of self-medication $\theta$ and vaccination $\nu$ on the spread of the disease is negligible — the value combinations of $\theta$ and $\nu$ for which $\mathcal {R}_t$ is above 1 corresponds to negligible values of $\nu$. We note that effective vaccination coverage is crucial in reducing the spread of the disease; self-medication plays a vital role in the spread of the disease in the event of little to no effective vaccination coverage — range of values of the proportion of the self-medicated population yielded $\mathcal {R}_t$ above 1 (see Fig. 8a, obtained by assuming an equal variation of $\theta$ across the different population groups).

Figure 8b-d show the effect of the proportion of self-medicated individuals in each population group and vaccination per capita on the effective reproduction number ($\theta _i$ vs $\nu$, $i=1,2,3$). We observe that among the age groups self-medication activities corresponding to age group 15-64 results in the highest value of $\mathcal {R}_t$ in the event of little to no effective vaccination coverage. Thus, this group should be a target for public campaign against self-medication. The effective reproduction number used here is the average for the entire period (from 1 to 127, consistent with case data used for parameter estimation) for each parameter value combination of $\theta$ and $\nu$. The computation process is outlined in Section 3 of the Supplementary Materials.

### Discussion

Self-medication and the use of complementary medicine is an integral component of disease treatment globally [1–6]. It is an alarming problem among resource limited countries in the global south. Even though self-medication has the potential of reducing health care expenditure [44], it has its own associated cost, among which, is the dampening effect it has on health policy interventions towards the control of infectious diseases. Self-medication as applied in our context of study can range from having faith that one will heal from the disease without ingesting any form of medicines to application of herbal medicine or over the counter drugs. Individuals who undergo self-medication in most cases do not use efficacious treatments. Not only does this increase the likelihood of prolonged infectious periods of the disease, it prevents isolation of these individuals as they do not make themselves available, therefore increasing the number of infectious individuals in the population, which will then amplify the force of infection within the population. As pointed out in [13], self-medication is a vital factor contributing to the spread of the disease although frequently overlooked; it contributes to the spread and severity of the disease and the population of individuals who under self-medication heightens the disease persistence against eradication.

This study proposed an age-structured mathematical disease model that incorporates self-medication in its dynamics; and used COVID-19 case data from Gauteng Province, South Africa, for analysis. We conducted uncertainty and sensitivity analysis on the model implied quantities—basic reproduction number, first epidemic peak, and first epidemic peak time—to the model parameters. The respective means of these quantities are 4.16499, 241,715, and 190.376. The model estimated proportion of individuals who self-medicated shows that self-medication is higher among age group 14-64 than the other age groups (0-14 and 65+). Also, the sensitivity analysis indicated that among the three age groups, age group 15-64 self-medicated activities has the most impact on the basic reproduction, first epidemic peak, and first epidemic peak time. Further analysis shows that self-medication is a vital factor impeding control of the disease in the absent of effective vaccination, however, has negligible joint impact on the disease with effective vaccination coverage. This we demonstrated by assessing the joint impact of the self-medication and vaccination on the average effective reproduction number. These findings show that in the case of Gauteng province, the active population (age group 15-64) have the highest level of self-medication incidence; (ii) self-medication is a crucial factor hindering control of the disease; (iii) self-medication joint impact with effective vaccination coverage on the spread of COVID-19 is negligible.

A weakness of our study is that, the proposed model used to address the research questions does not account for population demographics such as birth and death rates. Studies integrating this population demographics can provide that additional insight into addressing the research questions outlined in this paper. The method used to estimate the disease incidence cases for a given age group where such a group has no record cases assumes that cases are evenly distributed among the age groups. This assumption could either overestimate or underestimate the incidence cases in a representative group. Future work could address these gaps in our study.

## Conclusion

We addressed three research questions using Gauteng province, South Africa, COVID-19 cases spanning from the periods March 1, 2020 to July 5, 2020: (i) what is the impact of self medication across different age groups on the dynamics of the disease (example, disease prevalence)? (ii) what is the effect of the interplay of vaccination and self-medication on the spread of the disease? and (iii) which of the age groups has the highest incidence of self-medication? Using Gauteng province COVID-19 cases from the period March 1, 2020 to July 5, 2020, we have demonstrated that self-medication plays a crucial role in combating COVID-19, and that regardless of the level of effectiveness of instituted vaccination programs, it must be put in check. Appropriate campaign against COVID-19 related self-medication is justified. It is also worth noting that campaigns should target the active population (ages 14-64).

### Supplementary Information

Supplementary Material 1.

## Data Availability

The data is publicly available at: https://acadic.org/south-africa/.

## Abbreviations

ACADIC: Africa-Canada Artificial Intelligence and Data Innovation Consortium
ODEs: ordinary differential equations
MCMC: Markov Chain Monte Carlo
NMSE: Normalized mean square error
$\mathcal {R}_0$: Basic reproduction number
$\mathcal {R}_t$: Effective reproduction number
$S_i$: Susceptible population in age group *i*
$V_i$: Vaccinated population in age group *i*
$E_i$: Exposed population in age group *i*
$I_i$: infected population in age group *i*
$I^{sm}_i$: infected and self-medicating population in age group *i*
$I^{ft}_i$: infected population obtaining formal treatment in age group *i*
$V_i$: Removed population in age group *i*
$C_{i-j}$: Number of cases in age groups $i-j$
$P_{i-j}$: Population size of age groups $i-j$

## References

[1] Abdelwahed RNK, Jassem M, Alyousbashi A. Self-medication practices, prevalence, and associated factors among Syrian adult patients: a cross-sectional study. J Environ Public Health. 2022;2022:9274610. 10.1155/2022/9274610. 10.1155/2022/9274610 PMC9256391 35800339

[2] Awad A, Eltayeb I, Matowe L, Thalib L (2005). Self-medication with antibiotics and antimalarials in the community of Khartoum State. Sudan. J Pharm Pharm Sci..

[3] Osemene K, Lamikanra A (2012). A study of the prevalence of self-medication practice among university students in Southwestern Nigeria. Trop J Pharm Res..

[4] Omolase C, Adeleke O, Afolabi A, Ofolabi O (2007). Self medication amongst general outpatients in a Nigerian community hospital. Ann Ibadan Postgrad Med..

[5] Quincho-Lopez A, Benites-Ibarra CA, Hilario-Gomez MM, Quijano-Escate R, Taype-Rondan A (2021). Self-medication practices to prevent or manage COVID-19: A systematic review. PLoS ONE..

[6] Kamran A, Sharifirad G, Shafaeei Y, Mohebi S. Associations between self-medication, health literacy, and self-perceived health status: a community-based study. Int J Prev Med. 2015;6:66. 10.4103/2008-7802.161264. 10.4103/2008-7802.161264 PMC4521301 26288710

[7] Kazemioula G, Golestani S, Alavi SMA, Taheri F, Gheshlagh RG, Lotfalizadeh MH (2022). Prevalence of self-medication during COVID-19 pandemic: A systematic review and meta-analysis. Front Public Health..

[8] Makowska M, Boguszewski R, Nowakowski M, Podkowińska M. Self-medication-related behaviors and Poland’s COVID-19 lockdown. Int J Environ Res Public Health. 2020;17(22):8344. 10.3390/ijerph17228344 PMC7696561 33187315

[9] Quispe-Cañari JF, Fidel-Rosales E, Manrique D, Mascaró-Zan J, Huamán-Castillón KM, Chamorro-Espinoza SE (2021). Self-medication practices during the COVID-19 pandemic among the adult population in Peru: A cross-sectional survey. Saudi Pharm J..

[10] Sadio AJ, Gbeasor-Komlanvi FA, Konu RY, Bakoubayi AW, Tchankoni MK, Bitty-Anderson AM (2021). Assessment of self-medication practices in the context of the COVID-19 outbreak in Togo. BMC Public Health..

[11] Asamoah JKK, Owusu MA, Jin Z, Oduro F, Abidemi A, Gyasi EO (2020). Global stability and cost-effectiveness analysis of COVID-19 considering the impact of the environment: using data from Ghana. Chaos, Solitons Fractals..

[12] Eikenberry SE, Mancuso M, Iboi E, Phan T, Eikenberry K, Kuang Y, et al. To mask or not to mask: Modeling the potential for face mask use by the general public to curtail the COVID-19 pandemic. Infect Dis Model. 2020;5:293–308. 10.1016/j.idm.2020.04.001. https://www.sciencedirect.com/science/article/pii/S2468042720300117 10.1016/j.idm.2020.04.001 PMC7186508 32355904

[13] Kong JD, Tchuendom RF, Adeleye SA, David JF, Admasu FS, Bakare EA (2021). SARS-CoV-2 and self-medication in Cameroon: a mathematical model. J Biol Dyn..

[14] Liu K, Lou Y. Optimizing COVID-19 vaccination programs during vaccine shortages. Infect Dis Model. 2022;7(1):286–98. 10.1016/j.idm.2022.02.002. 10.1016/j.idm.2022.02.002 PMC8872681 35233475

[15] Mourad A, Mroue F, Taha Z (2022). Stochastic mathematical models for the spread of COVID-19: a novel epidemiological approach. Math Med Biol J IMA..

[16] Senapati A, Rana S, Das T, Chattopadhyay J (2021). Impact of intervention on the spread of COVID-19 in India: A model based study. J Theor Biol..

[17] Adiga A, Dubhashi D, Lewis B, Marathe M, Venkatramanan S, Vullikanti A. Mathematical models for covid-19 pandemic: a comparative analysis. J Indian Inst Sci. 2020;100(4):793–807. 10.1007/s41745-020-00200-6. 10.1007/s41745-020-00200-6 PMC7596173 33144763

[18] Shankar S, Mohakuda SS, Kumar A, Nazneen P, Yadav AK, Chatterjee K (2021). Systematic review of predictive mathematical models of COVID-19 epidemic. Med J Armed Forces India..

[19] Avusuglo W, Han Q, Woldegerima WA, Bragazzi NL, Ahmadi A, Asgary A, et al. COVID-19 and malaria co-infection: do stigmatization and self-medication matter? A mathematical modelling study for Nigeria. 2022. SSRN: https://ssrncom/abstract=4090040 or 10.2139/ssrn.4090040.

[20] Nasir M, Chowdhury A, Zahan T (2020). Self-medication during COVID-19 outbreak: a cross sectional online survey in Dhaka city. Int J Basic Clin Pharmacol..

[21] Niclós G, Olivar T, Rodilla V (2018). Factors associated with self-medication in Spain: a cross-sectional study in different age groups. Int J Pharm Pract..

[22] Department: Statistics South Africa RoSA. Mid-year population estimates. 2020. Accessed Nov 2021.

[23] Baracaldo-Santamaría D, Pabón-Londoño S, Rojas-Rodriguez LC (2022). Drug safety of frequently used drugs and substances for self-medication in COVID-19. Ther Adv Drug Saf..

[24] Brauer F, Castillo-Chavez C, Mubayi A, Towers S (2016). Some models for epidemics of vector-transmitted diseases. Infect Dis Model..

[25] Crokidakis N (2020). COVID-19 spreading in Rio de Janeiro, Brazil: Do the policies of social isolation really work?. Chaos, Solitons Fractals..

[26] Eikenberry SE, Mancuso M, Iboi E, Phan T, Eikenberry K, Kuang Y (2020). To mask or not to mask: Modeling the potential for face mask use by the general public to curtail the COVID-19 pandemic. Infect Dis Model..

[27] Hethcote HW (2000). The mathematics of infectious diseases. SIAM Rev..

[28] Tang B, Bragazzi NL, Li Q, Tang S, Xiao Y, Wu J (2020). An updated estimation of the risk of transmission of the novel coronavirus (2019-nCov). Infect Dis Model..

[29] Xue L, Jing S, Miller JC, Sun W, Li H, Estrada-Franco JG (2020). A data-driven network model for the emerging COVID-19 epidemics in Wuhan. Toronto and Italy. Math Biosci..

[30] Yousefpour A, Jahanshahi H, Bekiros S (2020). Optimal policies for control of the novel coronavirus disease (COVID-19) outbreak. Chaos, Solitons Fractals..

[31] Edholm CJ, Levy B, Spence L, Agusto FB, Chirove F, Chukwu CW (2022). A vaccination model for COVID-19 in Gauteng. South Africa. Infect Dis Model..

[32] Jacquez JA, Simon CP, Koopman J, Sattenspiel L, Perry T (1988). Modeling and analyzing HIV transmission: the effect of contact patterns. Math Biosci..

[33] Glasser J, Feng Z, Moylan A, Del Valle S, Castillo-Chavez C (2012). Mixing in age-structured population models of infectious diseases. Math Biosci..

[34] Haario H, Laine M, Mira A, Saksman E (2006). DRAM: efficient adaptive MCMC. Stat Comput..

[35] Laine M. 2018. https://mjlainegithubio/mcmcstat/#orgb0c2686. Accessed Nov 2021.

[36] Arregui S, Aleta A, Sanz J, Moreno Y (2018). Projecting social contact matrices to different demographic structures. PLoS Comput Biol..

[37] Prem K, Cook AR, Jit M (2017). Projecting social contact matrices in 152 countries using contact surveys and demographic data. PLoS Comput Biol..

[38] Prem K, Zandvoort Kv, Klepac P, Eggo RM, Davies NG, for the Mathematical Modelling of Infectious Diseases COVID-19 Working Group C, et al. Projecting contact matrices in 177 geographical regions: an update and comparison with empirical data for the COVID-19 era. PLoS Comput Biol. 2021;17(7):e1009098. 10.1371/journal.pcbi.1009098 PMC8354454 34310590

[39] Smith L, Hyndman R, Wood S. Spline Interpolation for Demographic Variables: The Monotonicity Problem. J Popul Res. 2004;21. 10.1007/BF03032212.

[40] Thomas SJ, Moreira ED, Kitchin N, Absalon J, Gurtman A, Lockhart S (2021). Safety and efficacy of the BNT162b2 mRNA Covid-19 vaccine through 6 months. N Engl J Med..

[41] Rothan HA, Byrareddy SN (2020). The epidemiology and pathogenesis of coronavirus disease (COVID-19) outbreak. J Autoimmun..

[42] Kirschner DE. Uncertainty and sensitivity functions and implementation. University of Michigan; 2007–2008. http://malthusmicromedumichedu/lab/usadata. Accessed 4 June 2024.

[43] Marino S, Hogue IB, Ray CJ, Kirschner DE (2008). A methodology for performing global uncertainty and sensitivity analysis in systems biology. J Theor Biol..

[44] Hughes CM, McElnay JC, Fleming GF (2001). Benefits and risks of self medication. Drug Saf..

