# Supplementary material for: Impact assessment of self-medication on COVID-19 prevalence in Gauteng, South Africa, using an age-structured disease transmission modelling framework
Source: BMC Public Health. 2024 Jun 7;24:1540. doi: 10.1186/s12889-024-18984-y (PMC11157731; doi:10.1186/s12889-024-18984-y)
Supplement: Supplementary file 1 — Supplementary Material 1. [file 12889_2024_18984_MOESM1_ESM.pdf]

Supplementary Materials

1. Model Equations

System 1 describes the evolution of the disease across the different compartments and age groups.

$$
\begin{aligned}
\dot{S}_i &= -\nu S_i - \mathcal{B}_i^s S_i,\\
\dot{V}_i &= \nu S_i - \mathcal{B}_i^v V_i,\\
\dot{E}_i &= \mathcal{B}_i^s S_i + \mathcal{B}_i^v V_i - \rho E_i,\\
\dot{I}_i^s &= \rho E_i - \alpha_i I_i - \mu_i I_i^s,\\
\dot{I}_i^{sm} &= \alpha_i \theta_i I_i - \eta_i^{sm} I_i^{sm},\\
\dot{I}_i^{ft} &= \alpha_i (1-\theta_i) I_i - \eta_i^{ft} I_i^{ft},\\
\dot{R}_i &= \mu_i I_i + \eta_i^{sm} I_i^{sm} + \eta_i^{ft} I_i^{ft},
\end{aligned}
\tag{1}
$$

with initial condition

$$Y(0) = (S_i(0), V_i(0), E_i(0), I_i^s(0), I_i^{sm}(0), I_i^{ft}(0), R_i(0)) \in \mathbb{R}^+.$$

1.1. Transmission rate $\mathcal{B}_i^s$ and $\mathcal{B}_i^v$

Let x_{ij} be the average number of contacts per person per unit time in a representative group, where $i = j$ is within group contact and $i \neq j$ outside group contacts. The unit time could be day(s) or month(s) (this study considered daily number of contacts). This defines the contact matrix in the population. We assumed heterogeneous effective transmission coefficients across age structures; these are respectively denoted as β_i^s and β_i^v for

susceptible and vaccinated individuals. $\mathcal{B}_i^s$ and $\mathcal{B}_i^v$ are expressed as

$$\mathcal{B}_i^s = \sum_{j=1}^{n} \frac{\beta_i^s x_{ij}(I_j + I_j^{sm})}{N_j},$$
$$\mathcal{B}_i^v = (1-e)\mathcal{B}_i^s, \tag{2}$$

where we note that

$$\beta_i^v = (1-e)\beta_i^s. \tag{3}$$

N_j is the population size of the individuals across age group j for all disease compartments, n is the number of age groups, and $0 \le e \le 1$ captures the vaccine efficacy.

2. Reproduction number derivation

Using the Next Generation matrix we derive the reproduction numbers as follows: We first write the vector T containing the rate of appearance of new infections in compartment c and Σ, the rate of transfer into and out of compartments c by all other means. Note that we the elements of T and Σ are the generalized representation of the model, thus we include the happenings in the vaccination compartments V_i. This is presented as

$$T_i = \begin{pmatrix} \mathcal{B}_i^s S_i + \mathcal{B}_i^v V_i \\ 0 \\ 0 \\ 0 \end{pmatrix}, \qquad \Sigma_i = \begin{pmatrix} \rho E_i \\ -\rho E_i + \alpha_i I_i + \mu_i I_i \\ -\alpha_i \theta_i I_i + \eta_i^{sm} I_i^{sm} \\ -\alpha_i (1-\theta_i) I_i + \eta_i^{ft} I_i^{ft} \end{pmatrix} \tag{4}$$

To begin with our derivation, we focus on the expression for the basic reproduction number. We hereby assume $\nu = 0$, and considered new infections

involving only susceptible compartment. We, therefore, have the matrix corresponding to T as

$$T^* = \begin{pmatrix}
0 & 0 & 0 & \frac{\beta_1^s X_{11} S_1(0)}{N_1} & \frac{\beta_1^s X_{12} S_1(0)}{N_2} & \frac{\beta_1^s X_{13} S_1(0)}{N_3} & \frac{\beta_1^s X_{11} S_1(0)}{N_1} & \frac{\beta_1^s X_{12} S_1(0)}{N_2} & \frac{\beta_1^s X_{13} S_1(0)}{N_3} & 0 & 0 & 0 \\
0 & 0 & 0 & \frac{\beta_2^s X_{21} S_2(0)}{N_1} & \frac{\beta_2^s X_{22} S_2(0)}{N_2} & \frac{\beta_2^s X_{23} S_2(0)}{N_3} & \frac{\beta_2^s X_{21} S_2(0)}{N_1} & \frac{\beta_2^s X_{12} S_2(0)}{N_2} & \frac{\beta_2^s X_{13} S_2(0)}{N_3} & 0 & 0 & 0 \\
0 & 0 & 0 & \frac{\beta_3^s X_{31} S_3(0)}{N_1} & \frac{\beta_3^s X_{32} S_3(0)}{N_2} & \frac{\beta_3^s X_{33} S_3(0)}{N_3} & \frac{\beta_3^s X_{31} S_3(0)}{N_1} & \frac{\beta_3^s X_{32} S_3(0)}{N_2} & \frac{\beta_3^s X_{33} S_3(0)}{N_3} & 0 & 0 & 0 \\
0 & 0 & 0 & 0 & 0 & 0 & 0 & 0 & 0 & 0 & 0 & 0 \\
0 & 0 & 0 & 0 & 0 & 0 & 0 & 0 & 0 & 0 & 0 & 0 \\
0 & 0 & 0 & 0 & 0 & 0 & 0 & 0 & 0 & 0 & 0 & 0 \\
0 & 0 & 0 & 0 & 0 & 0 & 0 & 0 & 0 & 0 & 0 & 0 \\
0 & 0 & 0 & 0 & 0 & 0 & 0 & 0 & 0 & 0 & 0 & 0 \\
0 & 0 & 0 & 0 & 0 & 0 & 0 & 0 & 0 & 0 & 0 & 0 \\
0 & 0 & 0 & 0 & 0 & 0 & 0 & 0 & 0 & 0 & 0 & 0 \\
0 & 0 & 0 & 0 & 0 & 0 & 0 & 0 & 0 & 0 & 0 & 0 \\
0 & 0 & 0 & 0 & 0 & 0 & 0 & 0 & 0 & 0 & 0 & 0
\end{pmatrix}.$$

and that of Σ is

$$\Sigma^* = \begin{pmatrix}
\rho & 0 & 0 & 0 & 0 & 0 & 0 & 0 & 0 & 0 & 0 & 0 \\
0 & \rho & 0 & 0 & 0 & 0 & 0 & 0 & 0 & 0 & 0 & 0 \\
0 & 0 & \rho & 0 & 0 & 0 & 0 & 0 & 0 & 0 & 0 & 0 \\
-\rho & 0 & 0 & \alpha_1 + \mu_1 & 0 & 0 & 0 & 0 & 0 & 0 & 0 & 0 \\
0 & -\rho & 0 & 0 & \alpha_2 + \mu_2 & 0 & 0 & 0 & 0 & 0 & 0 & 0 \\
0 & 0 & -\rho & 0 & 0 & \alpha_3 + \mu_3 & 0 & 0 & 0 & 0 & 0 & 0 \\
0 & 0 & 0 & -\alpha_1\theta_1 & 0 & 0 & \eta_1^{sm} & 0 & 0 & 0 & 0 & 0 \\
0 & 0 & 0 & 0 & -\alpha_2\theta_2 & 0 & 0 & \eta_2^{sm} & 0 & 0 & 0 & 0 \\
0 & 0 & 0 & 0 & 0 & -\alpha_3\theta_3 & 0 & 0 & \eta_3^{sm} & 0 & 0 & 0 \\
0 & 0 & 0 & -\alpha_1(1-\theta_1) & 0 & 0 & 0 & 0 & 0 & \eta_1^{ft} & 0 & \\
0 & 0 & 0 & 0 & -\alpha_2(1-\theta_2) & 0 & 0 & 0 & 0 & 0 & \eta_2^{ft} & 0 \\
0 & 0 & 0 & 0 & 0 & -\alpha_3(1-\theta_3) & 0 & 0 & 0 & 0 & 0 & \eta_3^{ft}
\end{pmatrix}.$$

We then numerically compute the $\mathcal{R}_0$ by noting that $\mathcal{R}_0$ is the spectral radius $T^*\Sigma^{*-1}$.

The matrices used to compute the effective reproduction numbers are Σ^* and

$$T_t^* = \begin{pmatrix}
0 & 0 & 0 & \frac{\beta_1^s X_{11} S_1(t)}{N_1} + (1-e)\frac{\beta_1^s X_{11} V_1(t)}{N_1} & \frac{\beta_1^s X_{12} S_1(t)}{N_2} + (1-e)\frac{\beta_1^s X_{12} V_2(t)}{N_2} & \frac{\beta_1^s X_{13} S_1(t)}{N_3} + (1-e)\frac{\beta_1^s X_{13} V_1(t)}{N_3} & \frac{\beta_1^s X_{11} S_1(t)}{N_1} + (1-e)\frac{\beta_1^s X_{11} V_1(t)}{N_1} & \frac{\beta_1^s X_{12} S_1(t)}{N_2} + (1-e)\frac{\beta_1^s X_{12} V_1(t)}{N_2} & \frac{\beta_1^s X_{13} S_1(t)}{N_3} + (1-e)\frac{\beta_1^s X_{13} V_1(t)}{N_3} & 0 & 0 & 0 \\
0 & 0 & 0 & \frac{\beta_2^s X_{21} S_2(t)}{N_1} + (1-e)\frac{\beta_2^s X_{21} V_2(t)}{N_1} & \frac{\beta_2^s X_{22} S_2(t)}{N_2} + \frac{\beta_2^s X_{22} V_2(t)}{N_2} & \frac{\beta_2^s X_{23} S_2(t)}{N_3} + (1-e)\frac{\beta_2^s X_{23} V_2(t)}{N_3} & \frac{\beta_2^s X_{21} S_2(t)}{N_1} + (1-e)\frac{\beta_2^s X_{21} S_2(t)}{N_1} & \frac{\beta_2^s X_{22} S_2(t)}{N_2} + (1-e)\frac{\beta_2^s X_{22} V_2(t)}{N_2} & \frac{\beta_2^s X_{23} S_2(t)}{N_3} + (1-e)\frac{\beta_2^s X_{23} V_2(t)}{N_3} & 0 & 0 & 0 \\
0 & 0 & 0 & \frac{\beta_3^s X_{31} S_3(t)}{N_1} + (1-e)\frac{\beta_3^s X_{31} V_3(t)}{N_1} & \frac{\beta_3^s X_{32} S_3(t)}{N_2} + (1-e)\frac{\beta_3^s X_{32} V_3(t)}{N_2} & \frac{\beta_3^s X_{33} S_3(t)}{N_3} + (1-e)\frac{\beta_3^s X_{33} V_3(t)}{N_3} & \frac{\beta_3^s X_{31} S_3(t)}{N_1} + (1-e)\frac{\beta_3^s X_{31} V_3(t)}{N_1} & \frac{\beta_3^s X_{32} S_3(t)}{N_2} + (1-e)\frac{\beta_3^s X_{32} V_3(t)}{N_2} & \frac{\beta_3^s X_{33} S_3(t)}{N_3} + (1-e)\frac{\beta_3^s X_{33} V_3(t)}{N_3} & 0 & 0 & 0 \\
0 & 0 & 0 & 0 & 0 & 0 & 0 & 0 & 0 & 0 & 0 & 0 \\
0 & 0 & 0 & 0 & 0 & 0 & 0 & 0 & 0 & 0 & 0 & 0 \\
0 & 0 & 0 & 0 & 0 & 0 & 0 & 0 & 0 & 0 & 0 & 0 \\
0 & 0 & 0 & 0 & 0 & 0 & 0 & 0 & 0 & 0 & 0 & 0 \\
0 & 0 & 0 & 0 & 0 & 0 & 0 & 0 & 0 & 0 & 0 & 0 \\
0 & 0 & 0 & 0 & 0 & 0 & 0 & 0 & 0 & 0 & 0 & 0 \\
0 & 0 & 0 & 0 & 0 & 0 & 0 & 0 & 0 & 0 & 0 & 0 \\
0 & 0 & 0 & 0 & 0 & 0 & 0 & 0 & 0 & 0 & 0 & 0 \\
0 & 0 & 0 & 0 & 0 & 0 & 0 & 0 & 0 & 0 & 0 & 0
\end{pmatrix}.$$

Now, for illustration purposes, we follow the works in [**?** **?**], and define $x_{ij} = c_i \gamma_{ij}$, such that c_i is the number of individuals contacted by an individual in group i per unit time (in our case per day) and γ_{ij} is the proportion of those contacts due to an individual in group i with individuals in group j. We note that $\sum_{i=1}^{n} \gamma_{ij} = 1$ and the matrix $\mathbb{P} = [(\gamma_{i,j})]$ is the mixing matrix. The mixing matrix determines the mixing pattern within and between the age groups. This mixing patterns can be defined under different assumptions: proportional mixing, restricted mixing, and preferential mixing [**?** **?**]. For the purpose of our study, we assume a proportional mixing process. This implies that γ_{ij} can be expressed as the proportion of total contacts attributable to the group j's population. We have

$$\gamma_{ij} = \frac{c_j N_j}{\sum_{j=1}^{n} c_j N_j} = \gamma_j.$$

By the above, the matrix $\mathbb{P}$ has it rows to be the same. For notational purpose, we set $x_{ij} = x_i = c_i \gamma_{ij} = c_i \gamma_j$ under this assumption. This implies that the product of $T_t^* \Sigma^{*-1}$ has rank 1. $\mathcal{R}_t$ is the summation of that of the

age specific groups. That said, from T_i and σ_i, we have

$$T_{ti}^* = \begin{pmatrix} 0 & \frac{\beta_i^s x_i S_i(t)}{N_i} + (1-e)\frac{\beta_i^s x_i V_i(t)}{N_i} & \frac{\beta_i^s x_i S_i(t)}{N_i} + (1-e)\frac{\beta_i^s x_i V_i(t)}{N_i} & 0 \\ 0 & 0 & 0 & 0 \\ 0 & 0 & 0 & 0 \\ 0 & 0 & 0 & 0 \end{pmatrix}$$

and

$$\Sigma_{ti}^* = \begin{pmatrix} \rho & 0 & 0 & 0 \\ -\rho & \alpha_1 + \mu_i & 0 & 0 \\ 0 & -\alpha_i\theta_i & \eta_i^{sm} & 0 \\ 0 & -\alpha_i(1-\theta_i) & 0 & \eta_i^{ft} \end{pmatrix}.$$

We can therefore obtain the expression for $T_i^*\Sigma^{*-1}$ and conclude that

$$\mathcal{R}_{ti} = x_i \left[\frac{S_i(t)\beta_i^s(\alpha_i\theta_i + \eta_i^{sm}) + V_i(t)\beta_i^v(\alpha_i\theta_i + \eta_i^{sm})}{N_i(\alpha_i + \mu_i)\eta_i^{sm}} \right],$$

where we note that $\mathcal{R}_{ti}$ is the effective reproduction number for age group i,$\beta_i^v = (1-e)\beta_i^s$, and

$$\mathcal{R}_t = \mathcal{R}_{t1} + \mathcal{R}_{t2} + \mathcal{R}_{t3}.$$

3. Computing effective reproduction number

The below algorithm computes the average value of the effective reproduction number for each value combination of θ and ν for periods from 1 to 127 days.

Algorithm 1 Estimating average value of effective reproduction number for each parameter value combination of θ and ν. Time period:1-127 in days.

1: Input baseline parameters and initial state values reported in Tables **??** and **??**

2: Set range of values for θ and ν

3: For each value of θ

    A. For each value of ν

        a. solve System Eq.1

            I For each time period

                i Compute effective reproduction number

            II Find the average effective reproduction number

4: End
